# Multicomponent fractionation of *Saccharina latissima* brown algae using chelating salt solutions

**DOI:** 10.1007/s10811-015-0785-0

**Published:** 2016-01-12

**Authors:** Martin Sterner, Ulrica Edlund

**Affiliations:** Fiber and Polymer Technology, KTH Royal Institute of Technology, Teknikringen 56, SE-100 44 Stockholm, Sweden

**Keywords:** *Saccharina latissima*, Kelp, Chelation, Extraction, Fractionation, Alginate

## Abstract

A fractionation strategy for *Saccharina latissima* algal biomass was developed utilizing chelating extraction salt solutions to mediate the liberation of algal components. Alginate, cellulose, laminarin, mannitol, protein, and inorganic salts were quantified in the fractions to reveal their individual dissolution patterns. Chelation power was identified as a key parameter for liberating alginate and increasing the yield of extracted components. The most efficient fractionation was achieved using aqueous sodium citrate as the extraction solution, producing an alginate-rich soluble fraction and a salt-poor insoluble fraction rich in cellulose and protein. Extractions at decreased pH were shown to be beneficial because they decreased the M/G ratio of the extracted alginate and concentrated the protein in the insoluble fraction from which it can easily be recovered; these effects could be achieved by switching the traditional sodium carbonate extraction solution with salts that have chelation capacity at lower pH. A cyclic extraction demonstrated that the sodium citrate solution can be reused for multiple alginate extractions with the buildup of the concentrations of other valuable components in the solution.

## Introduction

The continuous expansion of the agricultural sector has led to a situation where most suitable cultivable land is already being utilized. The desire to increase yields without the easy route of agricultural expansion provides incentive for the utilization of a greater part of the produced biomass. This separation and valorization of biomass fractions, sometimes regarded as waste streams, is referred to as biorefining and is a rapidly expanding field of science with strong societal interest (Kamm and Kamm 2004).

Recent agricultural expansion at sea is promising because it potentially affects the local environment less than terrestrial agriculture (Gao and McKinley 1994). Sea-based algal agriculture utilizes crops that live without the need for arable land, fresh water, or fertilization, circumventing three problems associated with land-based agriculture. Algae are grown commercially in many parts of the world, but upscaling comparable to land-based agriculture has yet to come (Zemke-White and Ohno 1999). To pave the way for an industry based on cultivated algae, specialized utilization of every part of the algae is important.

The brown algal species *Saccharina latissima* is known to thrive in the wild on the Swedish west coast, and preparations for large scale cultivation are currently underway (Karlsson 2007; Nielsen et al. 2014). *Saccharina latissima* stands out as a strong candidate biomass for future biorefinery initiatives due to its contents of a range of potentially valuable components with mono-, oligo-, and polymeric natures. Brown algae are commercially utilized for alginate production for use in the textile and food industries (McHugh 2003; Bixler and Porse 2011). However, alginate only accounts for approximately 20 % of the algal dry weight, and from a biorefinery perspective, efficient and straightforward pathways for the recovery and valorization of a substantial fraction of the algal components need to be developed and implemented (Jard et al. 2013).

The main components of *S. latissima* are alginate, laminarin, mannitol, cellulose, proteins, and salts (Pronin et al. 2012; Jard et al. 2013). Alginate, or alginic acid in its acid form, is an unbranched anionic polysaccharide comprised of the two uronic acids: mannuronic acid and guluronic acid. It serves as a structural support crosslinked by metal ions and bound to proteins (Larsen et al. 1970; Doubet 1982). Laminarin is a polysaccharide belonging to the group of glucans, and mannitol is a sugar alcohol. Proteins containing all essential amino acids and cellulose, which differs from wood cellulose in its crystal structure, are found as well (Atalla and Hackney 1991). A substantial part of the algal dry weight comprises inorganic salts, mostly sodium and potassium chlorides. In addition, alginate absorbs and retains polyvalent cations at concentrations significantly higher than those in the surrounding water (Percival 1979).

Many of the extractable constituents of *S. latissima* are susceptible to degradation when conventional extraction procedures are applied. For alginate, it is beneficial to retain as high of a molecular weight as possible during extraction because some applications, such as food and cosmetic formulations, require a high molecular weight products (Hernández-Carmona et al. 1999). In addition, in applications that allow for low viscosity alginate, less alginate would be required for a given function if the molecular weight was higher; for instance, viscosity modifiers for textile printing with 5 % low viscosity alginate can be replaced with 1.5 % high viscosity alginate (McHugh 1987).

Alginate degrades gradually, even in rather mild alkaline or acidic conditions (Haug et al. 1963). Still, the most common extraction procedure utilizes a solution of sodium carbonate (Na_2_CO_3_), which is a base salt that buffers a pH of approximately 11 at the concentrations needed but also degrades alginate more than a sodium hydroxide solution of equal pH (Haug et al. 1967a). A likely explanation for the extraction efficiency is the chelating effect of the carbonate ion (CO_3_^2−^) that is present under alkaline conditions. A chelating salt that operates at lower pH could keep the alginate intact, and it has been shown that chelating salts can extract alginate close to neutral pH (Rahelivao et al. 2013). Two factors known to improve the yield of alginate are increased extraction time and increased extraction temperature, however at the same time producing a more degraded alginate (Hernández-Carmona et al. 1999). If extraction at more neutral pH was possible, a higher temperature or longer extraction times could be tolerated without substantial degradation of the product.

Chelation, defined as coordinated ionic bonds to one ligand, provides greater ion affinity compared with mono-ionic bonds. Even though a few studies have tested chelators other than sodium carbonate for the extraction of alginate, there is still a lack of understanding of the impact of chelation. Several known effective chelators have been compared for the dissolution of alginate from algae, resulting in an almost complete extraction for all chelators, except for sodium carbonate performing slightly worse (Ahmad et al. 1993). The addition of the effective chelator EDTA to an alginate extraction with sodium carbonate increases the extraction yield slightly, also indicating that sodium carbonate is good, but not optimal, for extraction (Rahelivao et al. 2013). Two effective chelators, EDTA and CDTA, were compared in a study of extraction over time that yielded similar values for both (Wedlock et al. 1987). All of the performed extraction studies aimed for, and succeeded in, getting good extraction yields. At the same time, they gave little differentiable results on the yield and thus made it difficult to elucidate the impact of chelation strength.

Our aim was to unveil the role of chelation power in the liberation of alginate and essential components from brown algae under green conditions and develop a strategy for a first extraction step in a macroalgal biorefinery. Extraction solutions were chosen to cover a broad range of ion affinities using the association constant for calcium ion as a comparable measure of their individual strengths. To provide the foundation for a biorefining setup and to stake out routes for further fractionation, the major algal constituents (alginate, laminarin, mannitol, cellulose, protein, and salts) were measured in the extracted fractions. Mapping the solubilization of the remaining components after selective extraction of alginate also helps to visualize pathways, enabling the reuse of extraction solutions and concentration buildup in cyclic biorefinery processes.

## Experimental

**Fig. 1:**
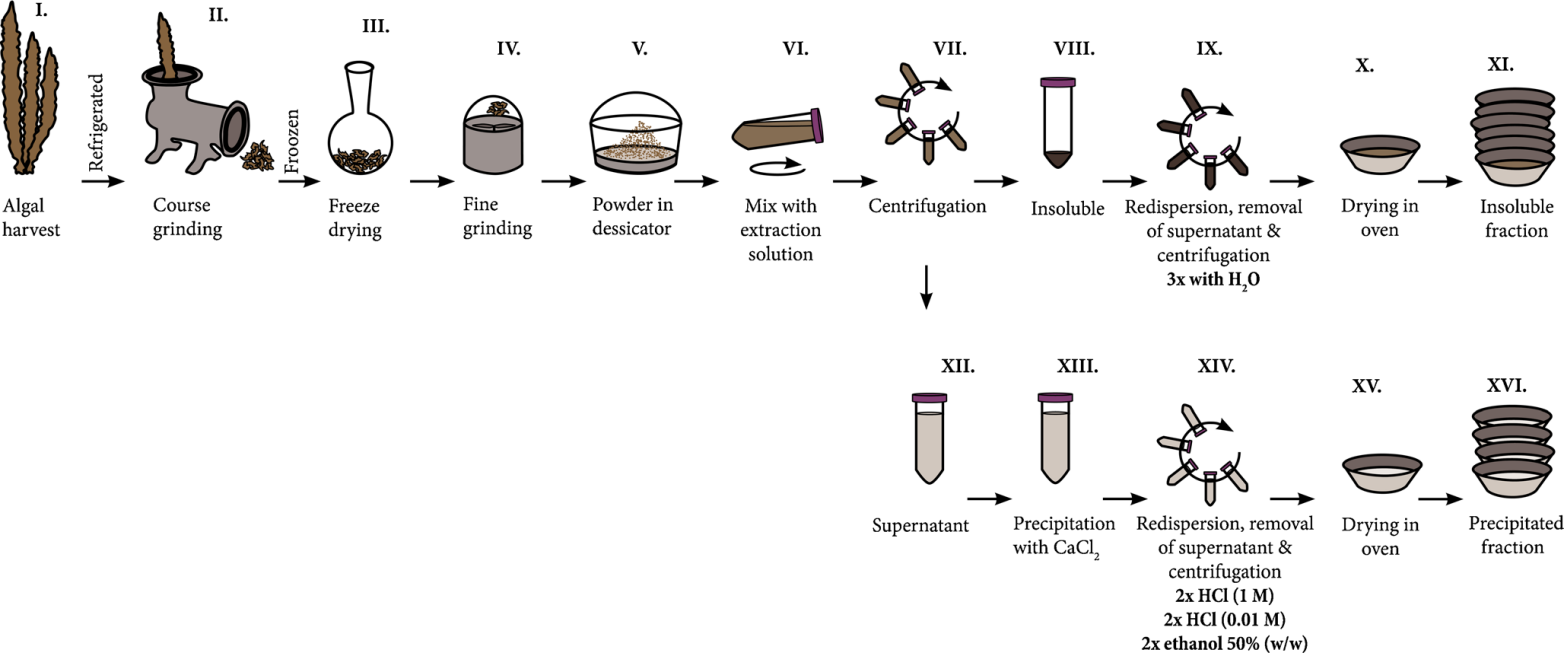
Experimental figure for the chelation driven fractionation of *Saccharina latissima* yielding an insoluble fraction (*XI*) and a soluble fraction (*XII*), which was subsequently precipitated by CaCl_2_ (*XVI*)

*Saccharina latissima* samples were collected on November 11, 2013, at Ursholmen (N 58° 50.124′, E 10° 59.418) by the Sven Lovén Centre for Marine Science (University of Gothenburg) on the Swedish west coast (Fig. 1, step I). The wet algae were refrigerated until they were coarsely ground in a Bruker meat grinder equipped with three consecutive hole plates with hole diameters of 45, 5, and 2 mm, respectively (Fig. 1, step II). The ground algae was frozen and kept at −20 °C until freeze drying (Fig. 1, step III). Freeze-dried algae was finely ground in an OBH Nordica Coffee Mill, Type 2393 (Fig. 1, step IV), and stored in a desiccator with dry silica gel (Fig. 1, step V) until further use.

Alginic acid from *Macrocystis pyrifera*, laminarin from *Laminaria digitata*, sodium succinate purum ≥98 %, sodium carbonate BioXtra ≥98 %, sodium oxalate ACS reagent ≥99.5 %, sodium citrate dihydrate ACS reagent ≥99 %, sodium acetate ≥99 %, sodium hydroxide ≥97 % ACS reagent, hydrochloric acid 37 % ACS reagent, l-(+)-arabinose ≥99, d-(+)-glucose ≥99.5 %, d-(+)-mannose ≥99 %, d-(+)-galactose ≥99 %, d-(+)-xylose ≥99 %, d-(+)-fucose ≥99 %, and nitric acid 70 % (*v*/*v*) ACS reagent were from Sigma-Aldrich. Sodium chloride ≥99.5 % was from Merck. d-(−)-Mannitol purum was from KEBO. Deuterium oxide (D_2_O) (DLM-4-100) 99.9 % was from Cambridge Isotope Laboratories, Inc. Sulfuric acid 72 % (*w*/*w*) was from Labservice. Ethanol 96 % (*v*/*v*) was from VWR. A multi-element solution (SS-028318) with 10 μM of Al, As, B, Ba, Ca, Cd, Co, Cr, Cs, Cu, Fe, Ga, Li, Mg, Mn, Na, Ni, Pb, and Zn in 3.6 % nitric acid was from Spectrascan.

### Main extraction procedure

The chelation driven fractionation procedure is schematically illustrated in Fig. 1, steps VI to XVI. Duplicate samples were prepared for every extraction.

#### Preparation of solutions and algal material

Eight different extraction solutions, 200 mL each, were prepared from deionized water (Table 1). The sodium carbonate and the sodium hydroxide solutions had starting pH values of 11.3 and 12.7, respectively. The pH of all other extraction solutions was adjusted to pH 9.3 ± 0.1 via titration with small amounts of sodium hydroxide.

**Table 1:** Composition of extraction solutions

Extraction solution	Chemical formula	Ion valency	Concentration [M]
Sodium citrate	Na_3_C_6_H_5_O_7_	3	0.1
Sodium carbonate	Na_2_CO_3_	2	0.15
Sodium oxalate	Na_2_C_2_O_4_	2	0.15
Sodium succinate	Na_2_C_4_H_4_O_4_	2	0.15
Sodium acetate	NaC_2_H_3_O_2_	1	0.30
Sodium chloride	NaCl	1	0.30
Sodium hydroxide	NaOH	1	0.30

#### Fractionation protocol

Samples were prepared in 50-mL falcon tubes to which 0.400 g algae powder, and 39.60 g of extraction solution were added followed by immediate mixing to homogenously disperse the algal powder. The falcon tubes were fixed horizontally on a shaking board with a shake frequency of 150 rpm and shake radius of 1 cm and left for 16 h at room temperature (Fig. 1, step VI).

After extraction, each falcon tube was centrifuged for 24 min at 4500 rpm with a rotational radius of 17 cm (Fig. 1, step VII).

After centrifugation, each supernatant was separated from the pellet and transferred to another 50-mL falcon tube (Fig. 1, steps VIII and XII). Calcium chloride (14 g, 1 M) was then added to each supernatant and allowed to react for 2 h to mediate precipitation (Fig. 1, step XIII).

#### Washing and drying of fractions

The insoluble pellet (Fig. 1, step VIII) was washed by adding 45 mL of water, vortexing, centrifugation, and removing the supernatant. This washing step was repeated three times (Fig. 1, step. IX).

The precipitates formed by the addition of calcium chloride (Fig. 1, step XIII) were first centrifugated, and the supernatant was discarded. Then, the samples were washed by adding 45 mL of different washing solutions, vortexing, centrifuging, removing the supernatant and then adding washing solution again. The first two washing cycles were performed with hydrochloric acid (1 M), followed by two cycles with hydrochloric acid (0.01 M), and, finally, two cycles with 50 % (*w*/*w*) ethanol in water (Fig. 1, step XIV). Because sodium oxalate forms a sparingly soluble calcium salt, it required five additional washing repeats with hydrochloric acid (Fig. 1, step XIV) to be visibly removed.

Purified samples from steps IX and XIV (Fig. 1) were transferred from falcon tubes to weighted alumina cups (Fig. 1, X and XV) and dried in a 65 °C oven for 3 days. The dry sample material in each cup (Fig. 1, XI and XVI) was then weighted and stored dry.

### Extraction with direct drying of fractions

In a parallel fractionation experiment, the extraction was performed as described in “Fractionation protocol” until reaching steps VIII and XII (Fig. 1). The supernatant (Fig. 1, step XII) and pellet (Fig. 1, step VIII) from each extraction were transferred from the falcon tubes to weighted petri dishes and dried. This protocol was applied to extractions using sodium hydroxide, sodium carbonate, sodium oxalate, and sodium citrate.

### Extraction of laminarin and mannitol

In an alternative extraction procedure, to extract laminarin and mannitol, the extraction was performed with sodium citrate as in “Fractionation protocol” until the alginate was separated (Fig. 1, step XIII). In this procedure, alginate was precipitated with hydrochloric acid (5 mL, 10 M) as the precipitant instead of calcium chloride, and the supernatant remaining after alginate removal was saved for further fractionation of laminarin and mannitol. To precipitate laminarin, acetone was added to give a 60 % (*v*/*v*) solution. Laminarin was isolated by centrifugation and purified by redispersion in fresh 60 % (*v*/*v*) acetone solution followed by centrifugation and drying of the pellet. The solution remaining after the recovery of laminarin was concentrated down to 20 mL and neutralized by addition of NaOH. To remove salt, acetone was again added to give a concentration of approximately 80 % (*v*/*v*). Salt was removed by centrifugation, and the supernatant containing mannitol was dried and stored.

### Extraction with reuse of extraction solvent

A cyclic extraction procedure was applied as an alternative route using hydrochloric acid as precipitant instead of calcium chloride (Fig. 1, step XIII). In these experiments, 0.425 g of algae was added to 42.1 g of sodium citrate solution. The insoluble pellet (Fig. 1, stepVII) was recovered as previously described (Fig. 1). The precipitation of alginate (Fig. 1, step XIII) was performed by adding 8.5 mL of hydrochloric acid (1.5 M) to 40 mL of the supernatant (Fig. 1, step XII). After 1 h of precipitation, the tubes were centrifuged and 45 g of the extraction solution was neutralized with 1.3 mL of sodium hydroxide (10 M) to reach a pH of 9.3–9.4. This regenerated extraction solution was first concentrated to approximately 39 mL and added to the next extraction cycle with a small amount of water to yield 42.1 g of extraction solution again. The extraction liquid was recycled three times.

### Characterization

#### Sample weight

Samples of each extracted fraction were weighed in alumina cups within 20 s after being withdrawn from a 65 °C oven. The alumina cups were previously conditioned in the same oven and weighed within the same time span.

#### Carbohydrate analysis

Carbohydrate analysis was performed via high-performance anion exchange chromatography (HPAEC) following the standard SCAN-CM 71:09 [SCAN-CM 71:09] with some modifications. Approximately 20 mg dry weight sample was subjected to acid hydrolysis, except for the low volume fractions when weights as low as 6 mg were used. Samples were soaked in 3 mL 72 % (*w*/*w*) of sulfuric acid for 1.5 h in 100-mL Pyrex flasks. The samples were manually grinded with a glass rod, and to decrease the oxygen, the flasks were put in a desiccator under vacuum atmosphere during the soaking. Deionized water (84 mL) was added to all flasks, yielding a concentration of 4 % (*w*/*w*) sulfuric acid when heated to 125 °C for 1.5 h in an autoclave. The carbohydrate compositions of the hydrolyzed samples were determined using a high-performance anion exchange chromatograph (Dionex, USA) equipped with a pulsed amperometric detector (HPAEC-PAD, Dionex ICS-3000) and CarboPac PA1 column (4 × 250 mm), using Milli-Q water and solutions of sodium hydroxide and sodium acetate. The eluent was pumped at 1.5 mL min^−1^ with a program starting with 0.05 M sodium hydroxide and increasing to 0.08 M sodium hydroxide with 0.190 M sodium acetate during the run. The data were processed with Chromeleon 7.1 software. The carbohydrate standards used for calibration were arabinose, galactose, glucose, xylose, mannose, and commercial alginate with a known mannuronic/guluronic acid composition. The composition of the reference alginate was determined by NMR. When the directly dried fractions (“Washing and drying of fractions”) were run, mannitol and fucose were used as standards.

#### Nuclear magnetic resonance (NMR)

NMR was used to determine the uronic acid composition of the alginate in the precipitated fraction (Fig. 1, step XVI). Commercial alginic acid from Sigma-Aldrich was used as a reference sample.

Samples were dissolved in water, and the pH was adjusted to 3 via small additions of dilute sodium hydroxide or hydrochloric acid. Then, samples were heated to 100 °C for 1 h. After cooling, the solutions were neutralized to pH 7 with sodium hydroxide, stirred until the alginate was completely dissolved, and then left to dry over 2 days at room temperature. Samples of approximately 2 % dried material were dissolved in deuterium oxide and transferred to NMR tubes with 5-mm diameters. ^1^H NMR spectra were recorded at 500 MHz on a Bruker DMX-500 NMR spectrometer. MestReNova software was used for data acquisition.

NMR analysis was also applied to extracted laminarin and mannitol using deuterium oxide as the solvent. Commercial laminarin and mannitol from Sigma-Aldrich were analyzed as reference samples.

#### Thermogravimetric analysis (TGA)

The moisture, char, and ash contents were determined according to the method provided by Anastasakis et al. (2011). Approximately 4 mg of extracted material from each fraction was tested. Each sample was heated from 40 to 800 °C at a rate of 25 °C min^−1^ under a nitrogen atmosphere with a 50-mL min^−1^ flow rate. At 800 °C, the atmosphere was changed to oxygen and the temperature was held constant for 20 min. The moisture content was calculated as the weight loss in the temperature interval from 40 to 110 °C. The char content was calculated as the weight remaining after heating from 110 to 800 °C under nitrogen atmosphere. The ash content was calculated as the weight remaining at the end of the heating program. Char% and ash% were calculated as percentages of the initial sample weight.

#### Protein analysis

Kjeldahl nitrogen analysis was performed at the Plant Nutrition Lab at the Department of Soil and Environmental Science, Swedish University of Agricultural Sciences. Samples containing 10 mL of supernatant from three extraction tubes (Fig. 1, step XII) were analyzed. In addition, 1 g of freeze-dried powdered algae was analyzed. The protein factor used for the calculation of the protein content from measured nitrogen content was 5.6 ± 0.4 (Bogolitsyn et al. 2014); this factor is derived to reflect the average amino acid composition of the entire algae, and because the proteins dissolved in the supernatant do not necessarily have identical compositions, the calculated protein values should be considered rough estimates.

#### Inductively coupled plasma optical emission spectroscopy (ICP-OES)

The metal ion contents of samples were measured using ICP-OES (iCAP-6000, Thermofisher AB).

Samples were taken directly from the dried supernatants and pellet fractions prepared as described in “Washing and drying of fractions” and material from 0.1 to 0.2 g dry algae was used for each sample. Prior to analysis, the samples were digested for 16 h in 30-cm-long borosilicate test tubes containing a mixture of 3 mL sulfuric acid 72 % (*w*/*w*) and 6 mL nitric acid 70 % (*w*/*w*) in heating blocks at 150 °C. After digestion, the samples were diluted 10 and 50 times for analysis and filtered through 0.45-μm cellulose filters from Millipore. Calibration standards were prepared from multi-element solutions with concentrations of 0, 0.5, 1.0, 5.0, and 7.5 μM for all the elements: Al, As, B, Ba, Ca, Cd, Co, Cr, Cs, Cu, Fe, Ga, Li, Mg, Mn, Na, Ni, Pb, and Zn.

#### pH measurements

A VWR SympHony SB70P pH meter equipped with a Hamilton Biotrode electrode was used to measure the pH of the extraction solutions before and after extraction. CertiPur® disodium hydrogen phosphate/potassium dihydrogen phosphate (pH 7.0) and CertiPur® potassium hydrogen phthalate (pH 4.01) solutions from Merck were used for calibration.

## Results and discussion

The brown alga *Saccharina latissima* grows fast and contains several potentially useful constituents that, if effectively extracted, could form the basis for a successful biorefinery. Alginate is in one sense the most sensitive component because its commercial value and applicability decrease if degraded during extraction or recovery. Hence, a viable fractionation route preferably starts with the extraction of alginate to preserve the structure because it is the major viscosity contributor. After alginate is removed, the remaining fraction can be repeatedly processed allowing for the concentration buildup of the other components. The most commonly used extraction agent, sodium carbonate, has the drawback that it operates at high pH, which is known to degrade alginate, providing motivation to find extraction media that operate at lower pH.

It is hypothesized that the chelation strength of polyvalent anions in the extraction solution toward calcium ions, calcium(II), is responsible for the solubilization of alginate in the algal cell wall. The multi-coordinated chelation bonds between alginate and divalent cations, referred to as an egg box structure, crosslinks alginate into a fiber network in the algal cell wall (Smidsrød and Grasdalen 1984). The dominant crosslinking ion in the alginate network is calcium because it is the most common polyvalent ion in the algae (Pronin et al. 2012). Hence, a chelating ion with a stronger affinity may facilitate calcium removal and aid in the dissolution of alginate.

In this work, a chelation-driven fractionation pathway is devised and the impact of the chelation of a set of sodium salts with varying chelation capacities was explored. Sodium citrate, sodium oxalate, and sodium carbonate were chosen as effective chelating salts, while sodium succinate was chosen because succinate is a polyvalent anion that is the same size as the effective chelators but with a low affinity for calcium ions. Sodium acetate and sodium chloride were chosen as reference salts because their monovalent anions cannot chelate at all. Extraction with deionized water was explored as a reference, and sodium hydroxide was used to reveal the effects of strongly alkaline conditions. Extraction with sodium hydroxide is commonly applied in the fractionation of other types of biomass, not in the least in processing of lignocellulosic material at high concentrations and temperatures (Gellerstedt 2009; Sjöström 2013).

All major fractions and components recovered were amply characterized with respect to composition, yields and structures to provide a knowledge basis for the potential application of chelation-based fractionation in a future biorefinery utilization of *S. latissima*.

### Extraction yields

**Fig. 2:**
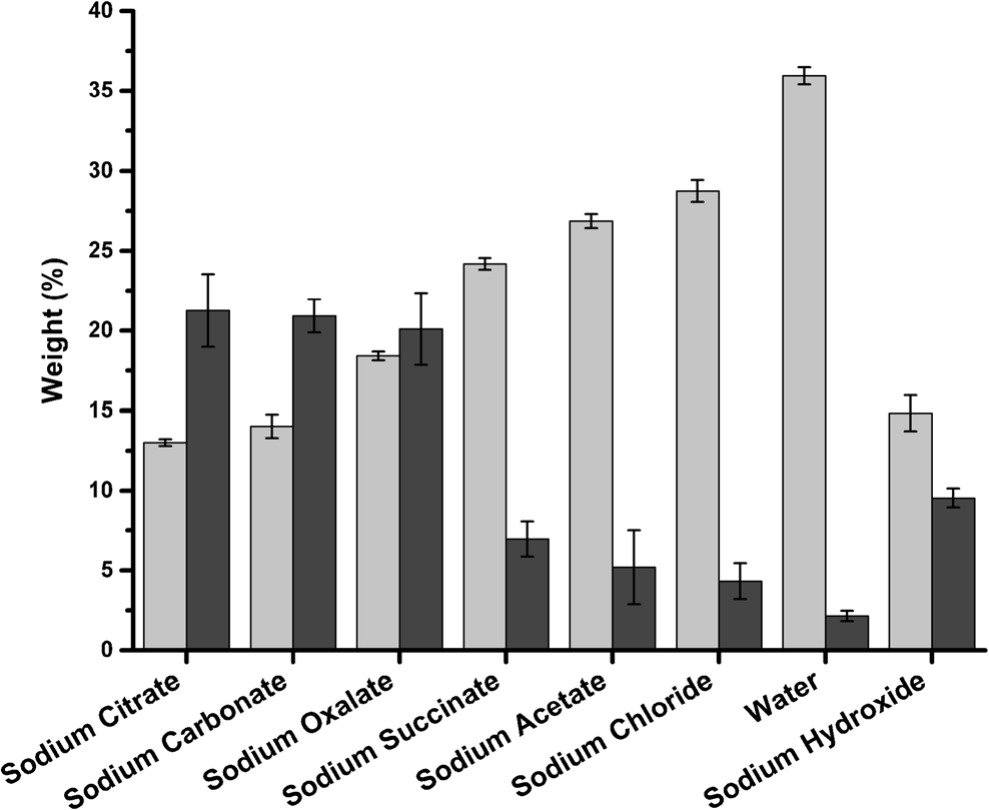
Yields of the insoluble fraction (*light gray*) and precipitated fraction (*dark gray*) after extraction with various solutions. Error bars are calculated as the T-distribution (95 %) confidence intervals

For all extraction solutions, the amount of Na^+^ was fixed at 0.3 M. The starting pH of the neutral extraction solutions was set to 9.3 ± 0.1 via the addition of a small amount of sodium hydroxide. The pH decreased during extraction, which indicates the release of acidic components from the algae (Table 2). The mass yields of insoluble and precipitated fractions (Fig. 1, steps XI and XVI) from extraction are summarized in Table 2 and Fig. 2.

**Table 2:** Mass yields from the insoluble and precipitated fractions (Fig. 1, steps XI and XVI) and pH in the extraction solution

		Sodium citrate	Sodium carbonate	Sodium oxalate	Sodium succinate	Sodium acetate	Sodium chloride	Water	Sodium hydroxide
Insoluble %(*w*/*w*)	Average	13.0 ± 0.16	14.0 ± 0.57	18.4 ± 0.21	24.2 ± 0.29	26.9 ± 0.34	28.8 ± 0.53	36.0 ± 0.42	14.8 ± 0.86
	T.DIST(0.95)	0.21	0.73	0.28	0.37	0.44	0.68	0.54	1.12
Precipitated %(*w*/*w*)	Average	21.3 ± 1.09	20.9 ± 0.50	20.1 ± 1.08	7.0 ± 0.53	5.2 ± 1.11	4.3 ± 0.54	2.2 ± 0.15	9.5 ± 0.29
	T.DIST(0.95)	2.278	1.039	2.251	1.102	2.314	1.119	0.321	0.611
pH	Before extraction	9.28	11.32	9.31	9.38	9.30	9.31	9.36	12.72
	After extraction	7.79	10.73	7.14	7.19	7.26	7.11	7.28	12.79

± indicates standard deviation. The number of sample duplicates was six for the insoluble fraction and four for the precipitated fraction. T.DIST(0.95) indicates the double sided T-distribution (95 %) confidence intervals

#### Structural and compositional analyses of the extracted fractions

High-performance anion exchange chromatography was used for the determination of uronic acids, monosugars, and sugar alcohol contents of the produced fractions. In the insoluble fractions, three sugar species were detected: guluronic acid, mannuronic acid, and glucose. The two former species verify the presence of alginate, whereas glucose indicates a high cellulose content in the insoluble fraction. Glucose is also a backbone building block in laminarin, a known component in brown algae; however, this polymeric species is more likely to accumulate in the dissolved fraction during extraction due to its high water solubility. The carbohydrate compositions of the insoluble fractions and the soluble/precipitated fraction are summarized in Table 3.

**Table 3:** Carbohydrate composition of the insoluble and precipitated fractions (Fig. 1, steps XI and XVI) recovered by chelation extraction

		Sodium citrate	Sodium carbonate	Sodium oxalate	Sodium succinate	Sodium acetate	Sodium chloride	Water	Sodium hydroxide
Insoluble	Glucose								
	mg g^−1^ sample	424 ± 47	391 ± 11	290 ± 33	161 ± 7	148 ± 24	137 ± 9	112 ± 9	277 ± 43
	mg g^−1^ dry algae	55 ± 6	55 ± 2	53 ± 6	39 ± 2	40 ± 7	39 ± 3	40 ± 3	41 ± 6
	Guluronic acid								
	mg g^−1^ sample	30 ± 12	32 ± 3	38 ± 10	145 ± 41	154 ± 21	163 ± 10	175 ± 15	129 ± 7
	mg g^−1^ dry algae	4 ± 1	4 ± 0	7 ± 2	35 ± 10	41 ± 6	47 ± 3	63 ± 5	19 ± 1
	Mannuronic acid								
	mg g^−1^ sample	70 ± 17	97 ± 4	72 ± 15	221 ± 63	238 ± 17	259 ± 2	285 ± 4	185 ± 21
	mg g^−1^ dry algae	9 ± 2	14 ± 1	13 ± 3	53 ± 15	64 ± 5	74 ± 1	103 ± 2	27 ± 3
	M/G ratio	2.33	3.08	1.92	1.53	1.55	1.59	1.63	1.43
Precipitated	Glucose								
	mg g^−1^ sample	10 ± 10	11 ± 10	12 ± 8	10 ± 9	27 ± 28	22 ± 21	63 ± 50	14 ± 10
	mg g^−1^ dry algae	2 ± 2	2 ± 2	2 ± 2	1 ± 1	1 ± 1	1 ± 1	1 ± 1	1 ± 1
	Guluronic acid								
	mg g^−1^ sample	316 ± 48	284 ± 31	310 ± 36	299 ± 37	396 ± 58	322 ± 37	429 ± 35	208 ± 24
	mg g^−1^ dry algae	67 ± 10	62 ± 6	59 ± 7	21 ± 3	21 ± 3	14 ± 1	9 ± 1	20 ± 2
	Mannuronic acid								
	mg g^−1^ sample	332 ± 2	459 ± 71	376 ± 2	243 ± 18	306 ± 52	261 ± 17	443 ± 18	397 ± 52
	mg g^−1^ dry algae	71 ± 0	76 ± 15	96 ± 0	17 ± 1	16 ± 3	11 ± 1	10 ± 0	38 ± 5
	M/G ratio	1.06	1.62	1.22	0.82	0.77	0.80	1.03	1.90
	Average sample weight (mg)	20.1	20.5	20.05	20	10	10.9	5.1	20.0
	Weight passing filter (mg)	17.8	18.0	18.5	18.8	7.7	8.5	3.0	17.0

± indicates standard deviation. The number of measured sample duplicates was two and the HPAEC results are the calculated means of two runs for each sample. Samples were placed in the reverse order for the second run

**Fig. 3:**
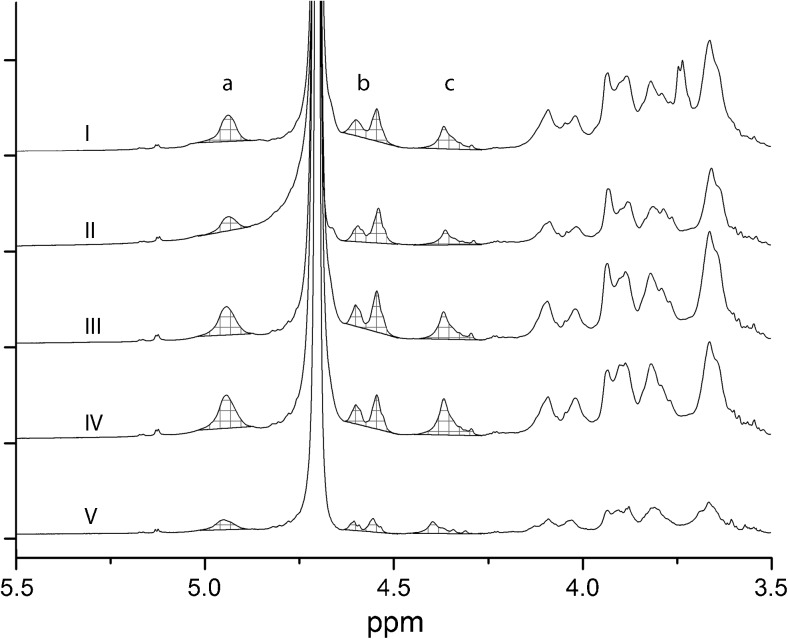
^1^H NMR spectra of precipitated fractions (Fig. 1, step XVI) using sodium citrate (*I*), sodium oxalate (*II*), sodium carbonate (*III*), sodium hydroxide (*IV*), and commercial alginic acid (*V*). The integrated peak areas *a*, *b*, and *c* were calculated between 5.02–4.88 ppm, 4.63–4.49 ppm, and 4.45–4.27 ppm, with a baseline between the start and the end point of integration as the lower constraint for the integration areas

The extraction solutions that gave lower insoluble fraction yields produced insoluble fractions that contained less mannuronic and guluronic acid from alginate with higher cellulose contents. These results indicate that the extraction solutions giving the lowest insoluble fraction yields, in particular sodium citrate, most effectively separate the polymeric components into separate fractions and allow for (i) more alginate to be recovered from the soluble fraction after precipitation with calcium chloride (Table 3) and (ii) a purer fraction of cellulose to be isolated (Fig. 3). Performing the extractions at elevated temperature would most likely increase the recovered yield of alginate (Hernández-Carmona et al. 1999) but also produce a more degraded alginate fraction.

Compositional data of the soluble fractions precipitated by calcium chloride (Fig. 1, step XVI) reveal a high content of uronic acid in all samples (Table 3). When the sample volume was varied, a smaller sample gave rise to a disproportionately high glucose signal. This result indicates that the measured glucose may be due to the effects of IC column contamination. The two extraction solutions operating at higher pH, sodium hydroxide and sodium carbonate, produced an alginate extract with a higher M/G ratio than that recovered during extraction with solutions at pH closer to neutral. This could be due to degradation of alginate at alkaline conditions and differences in solubility of the shorter fragments formed depending on their uronic acid composition (Haug et al. 1963, 1967b). Shorter fragments having mainly repeated MG sequence are more soluble in acidic conditions (Haug et al. 1967b). All other extraction solutions produced alginates with M/G ratios of approximately 1, and the variations were not statistically significant. In addition, the uronic acid composition was determined with ^1^H NMR for precipitated fractions (Fig. 1, step XVI) extracted with sodium citrate, sodium carbonate, sodium oxalate, and sodium hydroxide (Fig. 3). Commercial alginic acid from Sigma-Aldrich was used as a reference sample.

The uronic acid contents were calculated from the peak areas of a, b, and c in the ^1^H NMR spectra using Eqs. 1 and 2 (Grasdalen et al. 1979) and are given in Table 4:

1 $$ \mathrm{Percentage}\;\mathrm{of}\;\mathrm{guluronic}\;\mathrm{acid}=\frac{a}{b+c} $$

2 $$ \mathrm{Percentage}\;\mathrm{of}\;\mathrm{mannuronic}\;\mathrm{acid}=1-\frac{a}{b+c} $$

**Table 4:** Uronic acid composition determined using ^1^H NMR

	Sodium citrate	Sodium oxalate	Sodium carbonate	Sodium hydroxide	Alginic acid from Sigma-Aldrich
Guluronic acid (%)	45	51	38	29	39
Mannuronic acid (%)	55	49	62	71	61
M/G	1.21	0.95	1.60	2.41	1.55

The trend of higher M/G ratios with extractions performed at higher pH is seen in the results from both carbohydrate analysis and NMR. The results are also fairly similar between the methods with the difference in measured uronic acids being within 6 %. The NMR results for the M/G composition of the purchased alginate were used as references for the carbohydrate analysis with high-performance anion exchange chromatography. The trend that an extraction at lower pH gives a lower M/G ratio is interesting because a lower M/G ratio is preferable for many applications when a robust alginate gel is preferable, making stronger gels with the same amount of added calcium ions (Aarstad 2013). For comparison, in another study, an M/G ratio of 1.4 was detected for *Saccharina latissima* using a similar NMR methodology (Jard et al. 2013). The M/G ratio can be altered by the extraction method, making precise comparisons between studies difficult. The M/G ratio is well known to change with different growth conditions; for instance, the M/G ratio is known to increase as an adaptation to streaming water, giving increased flexibility to the algae (Draget et al. 2005).

**Fig. 4:**
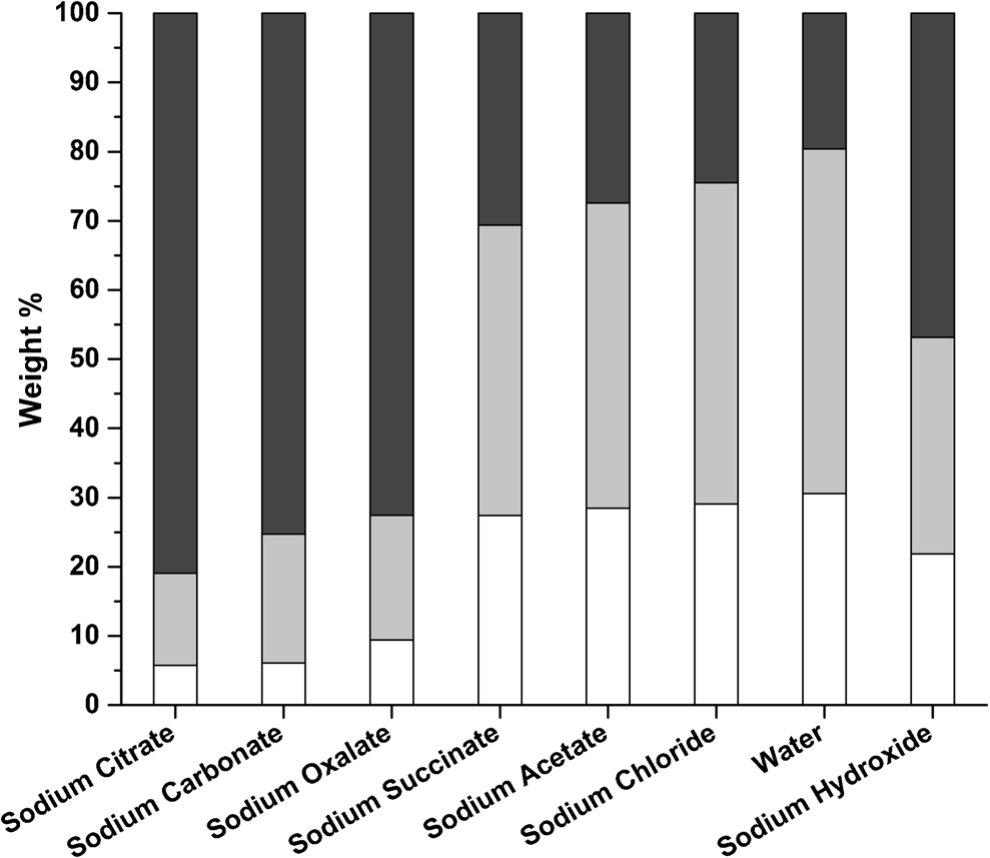
Relative contents of glucose (*dark gray*), mannuronic acid (*light gray*), and guluronic acid (*white*) in the insoluble fractions (Fig. 1, step VIII)

The measured uronic acid and glucose contents in the insoluble fraction do not add up to 100 % of the sample weight, indicating that additional algal components, other than alginate and cellulose, are recovered in the insoluble fraction and become soluble during the acid hydrolysis treatment prior to IC analysis. Inorganic salts and proteins are possibly accumulated in the unaccounted for fraction (Jard et al. 2013). The relative amounts of extracted glucose, mannuronic acid, and guluronic acid are given in Fig. 4, which visualizes the trends in the insoluble fraction carbohydrate contents independent of the other components.

In addition to cellulose and alginate, brown algae are reported to contain laminarin, a polysaccharide composed of glucose units (Kraan 2012). It is anticipated that laminarin, given its high water solubility, is collected in the soluble fraction during the chelation extraction described herein and further that the laminarin remains in the supernatant after alginate precipitation. To analyze the algal content of soluble species, a parallel extraction experiment was carried out using four of the extraction solutions (sodium citrate, sodium oxalate, sodium carbonate, and sodium hydroxide). In this extraction figure, both the soluble and insoluble fractions from each extraction were dried without further purification and subjected to acid hydrolysis and IC analyses according to the standard protocol previously described. Here, a wider array of sugar standards was used for the calibration of the IC allowing for the possible detection of mannitol and fucose in addition to the previously assessed carbohydrates. Glucose was indeed detected in all supernatants corresponding to a laminarin content of 220–250 mg g^−1^ dry algae (Table 5). Mannitol, ca. 100 mg g^−1^ algae, was also found in the supernatant fraction, and the detected fucose stems from the hydrolysis of fucoidan, a polyelectrolyte reported to be present in small amounts in brown algae (Bilan et al. 2010). Here, the fucose content is indeed very low, ca. 15 mg g^−1^ algae (Table 5). The insoluble pellets (Fig. 1, step VIII) were weighed before drying to calculate the percentage of supernatant that will be contained within the pellet during separation and remain in the insoluble fraction. This share of the supernatant fraction is given in Table 5 and has been subtracted from the insoluble fraction weights.

**Table 5:** Carbohydrate composition of the supernatant remaining after precipitation of the soluble fraction, and the insoluble fraction analyzed without purification

		Sodium citrate	Sodium oxalate	Sodium carbonate	Sodium hydroxide
Supernatant (mg g^−1^ dry algae)	Glucose (laminarin)	252	244	230	222
	Guluronic acid	75	88	63	26
	Mannuronic acid	104	109	111	61
	Fucose	16	15	14	14
	Mannitol	101	98	96	95
Insoluble (mg g^−1^ dry algae)	Glucose (cellulose)	53	54	46	46
	Guluronic acid	3	8	8	48
	Mannuronic acid	8	14	17	66
	Fucose	3	3	3	2
	Mannitol	2	1	1	1
Supernatant remaining in insoluble fraction (%)		6.5	4.3	6.5	8.8

Fucose and mannitol seem to be extracted in almost equal amounts regardless of the extraction solution used. The detected uronic acid contents differ slightly from the values recorded from the purified fractions, especially when sodium hydroxide was used as the extraction solution. Higher amounts of alginate are found both in the insoluble part and in the supernatant when additional purification was not applied. The results were compared with those of a seasonal variation study containing data from algal harvests from two consecutive years at a similar time of the year to that in this study, 7 October 2010 and 24 October 2011 compared with 11 November 2013 in this study (Schiener et al. 2015). The study was performed in Scotland at a similar latitude to the Swedish west coast, and the carbohydrate analysis was performed in a comparable way with similar sample hydrolysis and measurement techniques. The approximate average compositions were found to be alginate 20 %, laminarin 15 %, cellulose 15 %, and mannitol 20 % (Schiener et al. 2015). In comparison, the approximate composition found in this study is alginate 20 %, laminarin 25 %, cellulose 5 %, and mannitol 10 %. The alginate content was similar, while all other components differed significantly between the two studies. The big differences can be explained by the fact that so many parameters other than harvest time and latitude determine the composition, for instance, growth depth and water motion (Lüning 1979; Draget et al. 2005). The mannitol and laminarin contents change greatly during a season and are much lower during the summer season than the winter season. The laminarin content of 25 % in this study is comparatively high regardless of seasonal timing: The two reads in the Scottish study of approximately 15 % were the highest in the entire measurement series. The cellulose content of 5 % is also comparatively low regardless of seasonal timing, while the cellulose content was quite steady throughout the season in the Scottish study.

### Inorganic contents of the extracted fractions

The inorganic and char-forming contents of all fractions were assessed by TGA and are given in Table 6. The samples were first heated to 800 °C under nitrogen to quantify the char, and then, the atmosphere was changed to oxygen to detect ash. The moisture content was quantified as the weight loss from room temperature to 110 °C. All samples have low and comparable dry contents, facilitating comparisons without having to compensate for differences in water retention.

**Table 6:** Dry weight, char and inorganic contents of the insoluble and precipitated fractions (Fig. 1, steps XI and XVI) recovered by chelation extraction

		Sodium citrate	Sodium carbonate	Sodium oxalate	Sodium succinate	Sodium acetate	Sodium chloride	Water	Sodium hydroxide
Insoluble % (*w*/*w*)	Dry	94.5 ± 0.6	96.1 ± 1.0	96.8 ± 1.8	94.0 ± 0.9	93.7 ± 1.1	93.8 ± 0.9	93.1 ± 1.1	94.0 ± 0.8
	Char	28.8 ± 0.9	28.5 ± 1.3	30.1 ± 0.7	29.9 ± 0.3	29.5 ± 1.8	30.3 ± 0.7	31.7 ± 1.5	31.1 ± 1.4
	Ash	6.6 ± 1.1	10.1 ± 1.4	13.0 ± 2.4	12.0 ± 0.6	12.8 ± 1.3	12.9 ± 1.0	12.3 ± 1.0	14.9 ± 1.3
Precipitated % (*w*/*w*)	Dry	93.8 ± 0.8	95.0 ± 0.9	94.6 ± 1.7	95.0 ± 0.5	95.2 ± 0.5	95.0 ± 2.7	97.3	96.5 ± 0.9
	Char	28.3 ± 1.5	28.5 ± 1.4	26.8 ± 2.3	29.0 ± 1.2	27.5 ± 1.3	30.9 ± 4.6	27.1	28.4 ± 2.3
	Ash	6.4 ± 1.1	6.0 ± 0.9	4.8 ± 1.9	5.4 ± 2.0	5.2 ± 1.8	4.1 ± 2.7	1.8	3.3 ± 1.9

± indicates standard deviation. The number of measured sample duplicates was four for all samples except for the precipitated fractions with sodium succinate, sodium acetate and sodium chloride (three duplicates) and the precipitated fraction with water (one experiment)

It is reasonable to assume that the ash content in the insoluble fraction is related to the amount of alginate left in the fraction because polyvalent cations bind more strongly to alginate than to the other organic compounds present and because the insoluble fraction was washed three times with deionized water, which should have removed most monovalent ions that could otherwise form ash. The correlation between total ash and a higher alginate content is clear but not a linear relationship. For example, the insoluble fractions extracted using sodium citrate, sodium oxalate, and sodium carbonate all contain fairly similar amounts of alginate, but they have very different ash contents. To determine which polyvalent cations are present in the fractions, inductively couple plasma atomic emission spectroscopy (ICP-OES) was applied for the fractions prepared according to “Washing and drying of fractions,” where four extraction solutions were individually used (sodium citrate, sodium oxalate, sodium carbonate, and sodium hydroxide), and the supernatants remaining after precipitation of the soluble fraction were collected. The supernatants and the insoluble fractions from each extraction were dried without further purification, acid digested, and analyzed using ICP-OES. The results are summarized in Table 7. Calcium and magnesium constitute the major part of the polyvalent cations. Iron is the third most abundant ion and present in lower concentrations. From a biorefinery point of view, it is of particular importance to detect which fraction accumulates arsenic and what amounts, if any, are present in the algae due to the potential toxicity of arsenic species. Here, approximately 60 ppm is accumulated in the supernatant fraction, while trace amounts are detected in the insoluble fraction. The fact that the arsenic species are mainly collected in the supernatant phase in almost equal amounts regardless of the extraction solution used indicates that they consist of highly soluble anionic inorganic arsenites and arsenates that do not complex with the salt anions and are less affected by the different extraction solutions.

**Table 7:** Ash composition of the supernatants remaining after precipitation of the soluble fractions, and the insoluble fractions analyzed without purification

		Sodium citrate	Sodium oxalate	Sodium carbonate	Sodium hydroxide
Insoluble (ppm)	As	4	7	4	−1^a^
	Ca	739	3003	818	604
	Fe	114	85	99	112
	Mg	227	277	1200	3384
Supernatant (ppm)	As	64	59	62	68
	Ca	756	394	375	1396
	Fe	72	61	43	36
	Mg	4539	3781	3130	1372
Water solubility of salt composed of the divalent cation and the anion of the extraction solutions (g mL^−1^).	Ca^2+^	0.85	0.00067	0.00153	0.185
	Fe^2+^	Slightly soluble	0.022	0.0067	0.00015
	Mg^2+^	20.0	0.07	0.0106	0.0009

^a^Below detection limit

The relative amounts of calcium, iron, and magnesium in the supernatant and insoluble fraction are clearly deducible from the salt solubilities where a higher solubility results in higher relative amounts in the supernatant fraction. The sodium carbonate solution has a higher pH than the sodium citrate and sodium oxalate solutions, which noticeably affected the solubility of magnesium (Table 7). This effect is even more clearly pronounced when sodium hydroxide is used as the extraction solution. Extraction under highly alkaline conditions (in sodium hydroxide) also lowers the recovered arsenic content in the insoluble part.

### Estimated protein contents of the supernatant fractions remaining after precipitation of the soluble fraction

The protein contents (Table 8) of the supernatant fractions were estimated from the measured total Kjeldahl nitrogen using a conversion factor of 5.6, derived especially for *S. latissima* (Bogolitsyn et al. 2014). As a comparison, a sample of non-extracted dried algae was analyzed in parallel.

**Table 8:** Protein content, calculated from the total Kjeldahl nitrogen, of dry algae and supernatant fractions remaining after precipitation of the soluble fractions. pH of the extraction solutions after chelation extraction

	Algae	Sodium citrate	Sodium carbonate	Sodium oxalate	Sodium succinate	Sodium acetate	Sodium chloride	Water	Sodium hydroxide
Protein % (*w*/*w*), dry algae	7.6	3.0	4.0	2.7	2.3	2.4	2.4	2.6	6.1
pH at extraction	Prior to extraction	7.79	10.73	7.14	7.19	7.26	7.11	7.28	12.79

The values presented in Table 8 indicate that pH is an important factor for the extraction of proteins from algae. While the extraction solutions operating at neutral pH produce supernatants with protein contents in the 2.3–3 % *w*/*w* range with minor variations, sodium carbonate and, even more so, sodium hydroxide release more protein to the supernatant phase. These results are in line with protein extraction studies on other materials (Nolsøe and Undeland 2009). This could also help explain why more arsenic was detected in the supernatant fractions extracted at higher pH as previously discussed (Table 7). Inorganic arsenic species are known to bind strongly to proteins (Shen et al. 2013), and a higher protein content may capture the arsenic species more effectively. It should be noted that the protein conversion factor of 5.6 is derived for the calculation of the protein contents in intact algae. When applied to extracts rather than algal tissue, this N factor merely provides an estimate. The total amount of protein is reasonable and can be considered a normal protein content for *S. latissima* (Bogolitsyn et al. 2014).

### Chelation strength and association constant for calcium ions

Calcium is the most abundant polyvalent cation in seawater and possesses a strong affinity for alginate, differentiating it from magnesium, which is the second most abundant polyvalent cation. Hence, calcium ions have by far the strongest impact on the recovered alginate structure and solubility. In the chelation extraction protocol proposed here, the efficiency of the chelating ion in separating alginate from the algal tissue may be understood on the basis of chelating ion–calcium affinity. The association constant of each of the studied anions to Ca^2+^ may serve as a model for quantifying this affinity: If the anion–calcium affinity is high, the formation of such bonds competes with the ionic bonds between calcium and the alginate uronic acids. If calcium is transferred from alginate to the chelating salt, the alginate will become increasingly soluble and may be released from the algal tissue to the soluble fraction during extraction.

Chelation is a special case of ion affinity where coordinated ions bind to one ligand and give far larger dissociation constants than mono-ionic bonds. This difference between monovalent anions and polyvalent anions is easily seen when the association constants to Ca^2+^ for the anions in the extraction solutions are compared (Table 9) (Martell and Smith 1974). Anions with two or more carboxylic acid groups display higher association constants than monovalent anions. The citric acid ion, which has three carboxylic moieties, has the highest calcium association constant among the anions studied. Indeed, sodium citrate is the most effective extraction solution among the studied systems and produces the purest cellulose fraction and the highest yield of laminarin in the supernatant along with the most efficient separation of alginate from the other algal components allowing for the recovery of alginate in the precipitated fraction.

**Table 9:** Calcium ion association constants to various anions (Hawley 1973; Martell and Smith 1974) for the formation of ion complexes at the listed ionic strengths and adjusted to the ionic strength of the extraction (calculated by Eq. 5)

	Citric	Carbonic^a^	Oxalic^b^	Succinic	Hydroxide	Acetic	Chloric
Ion valency	−3	−2	−2	−2	−1	−1	−1
Log Ca^2+^ association constant in the literature reference	3.45	2.21	2.46	1.25	1.0	0.57	−0.14
Ionic strength in the literature reference	0.1	0.72^c^	0.1	0.1	0.1	0.1	1^c^
Ionic strength at extraction	0.6^c^	0.45	0.45	0.45	0.3	0.3	0.3
Log Ca^2+^ association constant at ionic strength of extraction	2.72	2.28	2.03	0.82	0.83	0.40	−0.06

^a^No association constant in proximity to the ionic strength at extraction was found for the carbonate ion in Martell, and Smith critical stability constants, and thus, a value from another publication was used

^b^Value obtained at 37 °C instead of 25 °C as for all other values

^c^The Davies equation, used for the calculation of the calcium ion association constant at the ionic strength of the extraction, is developed for ionic strengths below 0.5 but can be used above with less reliability

**Fig. 5:**
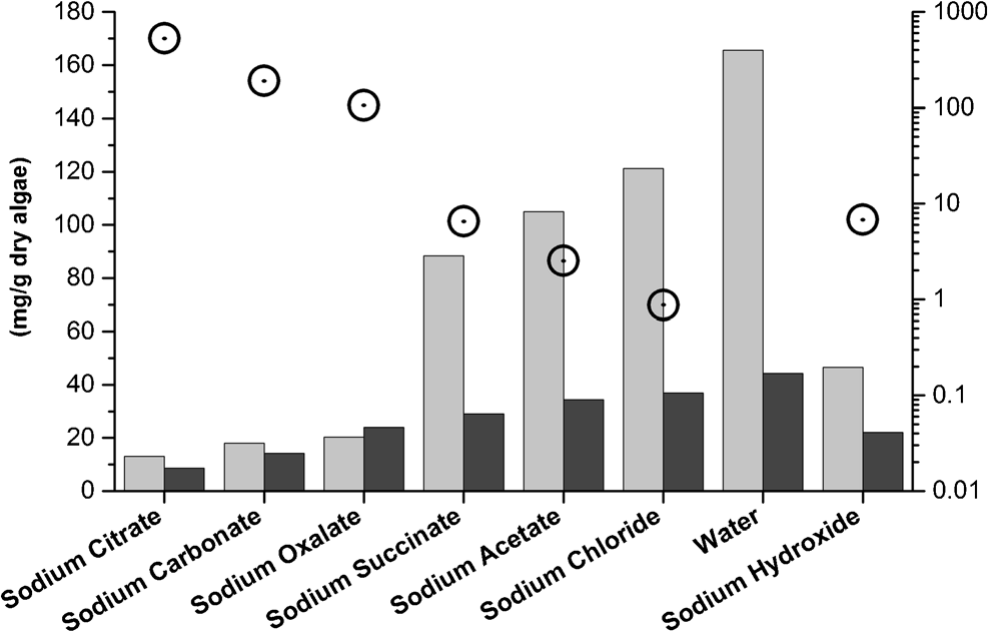
Alginate (*light gray*) and ash (*dark gray*) contents of the insoluble fractions and Ca^2+^ ion association constants of the anions in each extraction solution (*circles*), which follows the logarithmic scale to the right

The superior efficacy of the sodium citrate extraction system is further demonstrated by comparing the calcium ion association constants with the remaining amounts of ash and alginate in the insoluble fraction (Fig. 5). The chelating salts with higher affinities to calcium also provide a more pronounced solubilization of alginate increasing the alginate yield upon recovery from the soluble fraction by precipitation. The same trend applies to the release of inorganic compounds and laminarin from the algae to the soluble fraction during extraction (Table 5).

The chelation strength of a chelator is dependent on the ionic strength of the solution. The empirical Davies equation (Eq. 3) allows for estimation of the activity coefficient of an ionic interaction at the given ionic strength in the solution (Güntelberg 1926). This is further used as an activity correction for the two component equilibrium (Eq. 4) to estimate the association constant between the two ions at zero ionic strength (*K*_ass_I0_). Combining Eqs. 3 and 4 gives Eq. 5, which can be used to estimate the activity constant *K*_ass_ at ionic strengths other than the given one. Ionic strength is sometimes listed in the literature as the zero ionic strength association constant, which can be used to estimate the value of the association strength at a given ionic strength. The zero ionic strength association constant can be measured in solutions with very low ionic strengths, but it is hard to approximate a value at higher ionic strengths, especially if the valences of the involved ions are >1 (Martell and Smith 1974). A better way than using the listed zero ion strength association constants for approximations at high ionic strength is to find a measured association constant value at an ionic strength closer to the ionic strength of interest and begin the approximation from that value. In that case, the adjustments to the measured value get smaller, and thus, the room for error is less pronounced.

*z*_1,2_: Charges of the cation and anion that are interacting
*ƒ*_±_: Activity coefficient
*I*_1,2_: Two different ionic strengths
$$ {K}_{{\mathrm{ass}}_{1,2}} $$
Association constants at two different ionic strengths
$$ {K}_{\mathrm{ass}\_{I}_{\mathrm{O}}} $$
Association constant at zero ionic strength

3 $$ - \log \left({\mathrm{f}}_{\pm}\right)=0.5{z}_1{z}_2\left(\frac{\sqrt{I}}{1+\sqrt{I}}-0.15\;I\right) $$

4 $$ {K}_{\mathrm{ass}\_{I}_{\mathrm{O}}}=\frac{K_{\mathrm{ass}}}{{\left({\mathrm{f}}_{\pm}\right)}^2} $$

5 $$ {K}_{{\mathrm{ass}}_2}={K}_{{\mathrm{ass}}_1}\kern0.5em \times \kern0.5em {10}^{z_1{z}_2\kern0.5em \times \kern0.5em \left(\frac{\sqrt{I_1}}{1+\sqrt{I_1}}-\frac{\sqrt{I_2}}{1+\sqrt{I_2}}+0.15\left({I}_2-{I}_1\right)\right)} $$

#### Chelation extraction at higher ion strength and with recycling of the extraction solution

Alginate contributes to a high viscosity of the soluble fraction and thus sets the limit for the algae:solution ratio during extraction. However, from a biorefinery and sustainability perspective, it would be attractive to minimize the volume of the extraction solution used. Hence, the reuse of extraction solution in consecutive extractions stands out as an interesting alternative. After removal by precipitation of alginate from the extraction solution, the remaining solution could be reused for the dissolution of the next batch without increasing the viscosity. However, if the solution is to be reused, the use of calcium chloride for alginate precipitation is not applicable because the residual content of solubilized calcium would then inhibit further alginate dissolution in the next extraction cycle. Instead, hydrochloric acid was added for the precipitation of alginate, allowing the solution to be neutralized with sodium hydroxide and reused after the alginate recovery. The yields, ash, and salt contents in the reused extraction solution are summarized in Table 10. After every cycle, more salt will accumulate in the extraction solution and decrease the calcium ion association constant, which sets a practical limit to the number of reuse cycles that can be implemented. The yield of the insoluble fraction increased for each new extraction cycle, which could indicate a gradual loss of chelating ion efficiency for alginate extraction to the supernatant fraction. The ash content was small and unlikely to impact the extractions to any significant extent.

**Table 10:** Multiple extractions with reuse of the extraction solution

	Yield % (*w*/*w*) insoluble	Yield % (*w*/*w*) precipitated	Ash % (*w*/*w*) insoluble fraction	Ash % (*w*/*w*) precipitated fraction	Accumulated 1salt in solution	Ionic strength
First cycle	13.1 ± 0.03	18.5 ± 0.04	1.15	0.75	0 M NaCl	0.6
Second cycle	13.9 ± 0.23	19.7 ± 0.00	0.78	0.60	0.31 M NaCl	0.9
Third cycle	14.8 ± 0.03	18.3 ± 0.83	1.04	0.78	0.62 M NaCl	1.2

± indicates standard deviation. The number of sample duplicates was two

## Conclusions

The chelation power of the extraction solution was found to be a key parameter in a multicomponent fractionation pathway designed for the liberation of components from the brown algae *Saccharina latissima*. The extraction pathway produced fractions with alginate, laminarin, mannitol, cellulose, protein, and salt that were quantified and characterized. The gross carbohydrate composition of the algae was alginate 20 %, laminarin 25 %, cellulose 5 %, and mannitol 10 %.

With increasing chelation power of the extraction solutions, alginate was more efficiently isolated and the yield increased. Among the extraction solutions explored, sodium citrate was most efficient, operating at slightly alkaline conditions and producing the purest alginate fraction with an M/G ratio of 1.1 and a cellulose-rich insoluble fraction. The uronic acid composition of alginate was affected by the extraction pH. Solutions operating at higher pH values, such as sodium citrate, increased the preferable guluronic acid content in the recovered alginate.

The dissolution of sparingly soluble salts also increased with increasing chelation power and was highest when applying sodium citrate as the extraction solution, while the other strong chelators, i.e., sodium carbonate and sodium oxalate, formed salt precipitates with the most abundant polyvalent cations. The protein yield was not affected by chelation strength; instead, solubilization increased with increased pH of the extraction solution. Laminarin and mannitol were less affected by variations in the extraction parameters. However, the highest yield was still achieved using sodium citrate-mediated extraction.

Chelation power was quantified as the association constant between the polyvalent anion of the extraction salt and a calcium(II) ion because calcium is the most abundant polyvalent cation and is structurally integrated into the algal cell wall. The good correlation between the association constant and the recorded extraction yields of alginate and the sparingly soluble salts indicated that the chelation strength model was relevant. Extraction with sodium citrate dissolved most of the sparingly soluble salts, a result that is important for the recovery of salt-deficient cellulose and protein fractions that can have further uses in biorefineries.

The concept of a cyclic biorefinery system for the recovery of alginate and the concentration buildup of other soluble constituents was successfully achieved with sodium citrate as the chelating agent in the extraction solution. This system could enable the utilization of the highly soluble compounds laminarin and mannitol, which can be recovered after their concentrations become sufficiently high, giving flexibility to a biorefinery that has to handle biomass with high compositional variations.
